# Management of Peri-Implant Diseases: A Survey of Australian Periodontists

**DOI:** 10.3390/dj8030100

**Published:** 2020-09-01

**Authors:** Ahsen Khan, Dileep Sharma

**Affiliations:** 1 College of Medicine and Dentistry, James Cook University, 14-88 McGregor Road, Smithfield 4878, QLD, Australia; ahsen.khan@my.jcu.edu.au; 2 Department of Periodontics, James Cook University, 14-88 McGregor Road, Smithfield 4878, QLD, Australia; 3 The Australian Institute of Tropical Health and Medicine (AITHM), James Cook University, 14-88 McGregor Road, Smithfield 4878, QLD, Australia

**Keywords:** peri-implant mucositis, peri-implantitis, dental implants, therapeutics, aetiology

## Abstract

Background/Aim: This survey-based study aims to explore the clinical management protocols of followed by Australian periodontists in relation to peri-implant diseases. Materials and Methods: A five-part online questionnaire was developed and administered through email. Descriptive statistics were used for analysis, with the univariate associations between a categorical outcome and the variables evaluated using Pearson’s Chi-squared test. Results: The survey yielded 99 responses, resulting in a response rate of 41.8%. Most participants were male and aged 35–44 years. More than a quarter of practitioners had been placing implants for 6–10 years and almost two-fifths of practitioners placed 1–10 implants per month. The estimated prevalence of peri-implant mucositis and peri-implantitis in the general Australian population was 47% and 21%, respectively. Practitioners reported using systemic antibiotics to manage peri-implant mucositis (7%) and (72%) peri-implantitis lesions, with a combination of amoxicillin and metronidazole. Most common treatment modalities were oral hygiene instructions, nonsurgical debridement and antimicrobial gel/rinse. Surgical debridement and systemic antibiotics were also often used for peri-implantitis treatment. Practitioners preferred a 3-month clinical follow-up and 6-month radiographic evaluation. Furthermore, three-quarters of practitioners rated their management as moderately effective, although upwards of nine-tenths expressed the need for further training and awareness. Conclusion: This study confirms a significant use of empirical treatment modalities due to lack of standard therapeutic protocol. However, some approaches followed by the specialists may provide a basis to formulate a therapeutic protocol for peri-implant disease management.

## 1. Introduction

The advent of osseointegrated dental implants has revolutionised the treatment of edentulous patients over the past four decades [1,2,3,4,5]. High success rates that dental implant therapy achieves in various clinical situations has resulted in implant survival rate of 82.6% up to 15 years [4]. Despite this, dental implants are still subject to several complications which can compromise this high success rate [1,2,3,4]. Adler et al. reports that biological complications are the most common complication, responsible for over half (52%) of all encountered complications [4]. These complications include peri- implant mucositis and peri-implantitis, both of which are inflammatory diseases associated with the placement of dental implants [1,2,3,4,6,7].

In 2017, the ‘World Workshop on Periodontology’ (WWP) defined peri-implant mucositis as “an inflammatory lesion of the soft tissues surrounding an endosseous implant, in the absence of loss of supporting bone or continuing marginal bone loss [6].” Further, peri-implantitis was defined as “a pathological condition occurring in tissues around dental implants, characterized by inflammation in the peri-implant connective tissue and progressive loss of supportive bone [7].”

Prevalence of peri-implant mucositis has been reported to be close to 47% whilst peri-implantitis occurred in 20% of implant patients [8]. Prevention and successful treatment of peri-implant diseases relies on a thorough understanding of the aetiological factors implicated in the pathological condition. A definitive cause-and-effect relationship between bacterial biofilms and peri-implant mucositis has been long established [6]. This is critical as peri-implant mucositis is considered a precursor for peri- implantitis, although the histopathologic and clinical conditions driving this progression are not yet completely understood [6,7]. In addition to bacterial accumulation, several other aetiological factors and risk indicators have been identified in relation to peri-implant diseases, including, poor oral hygiene, history of periodontitis, excess cement, smoking, systemic conditions (i.e., diabetes mellitus), absence of keratinized mucosa, improper design of implant-supported restorations and occlusal overloading [6,7,9].

Despite a growing knowledge base around peri-implant pathologies, the treatment of such conditions remains a clinical challenge. This is primarily due to the lack of evidence-based guidelines available to clinicians in utilising different treatment modalities. Currently, a gold-standard therapeutic protocol for peri-implant pathological lesions does not exist [10,11,12,13].

Dental implant treatment has risen significantly worldwide over the last few decades, with Australia being described as a “fast expanding market” [6]. Guo et al. also noted that an increasing proportion of dental implant treatment is being carried out by general dental practitioners, so much so that they predicted that “implant dentistry will become a significant part of contemporary general dental practice in the foreseeable future” [6]. Prevalence of peri-implant pathologies has also been shown to be positively correlated with the duration for which the implant is in function [8]. Based on both these factors, it can be projected that biological failures will further affect implant success rates in Australia in the near future.

In 2009, Mattheos et al. surveyed and compared Australian periodontists to British periodontists in relation to their management decisions and attitudes towards peri-implantitis [2]. Their study concluded that neither country had a universal approach to managing peri-implant pathologies, with significant heterogeneity being reported [2]. Similar heterogeneity was reported when comparable studies were conducted in Sweden [14] and the United States of America [1] (USA).

With an ever-increasing number of implants being used to rehabilitate patients in Australia and those implants being functional units for a greater number of years, it is imperative that a well-defined evidence-based clinical protocol needs to be adopted so as to guide clinicians treating biological complications of implant therapy. In recent years, there has been a concerted effort to increase understanding of peri-implant pathologies, including definitions and diagnostic tools [3,6,7].

This study was conducted to investigate the perceived prevalence along with the aetiological factors for peri-implant pathologies, and to investigate the therapeutic protocols most commonly used to manage these conditions by Australian periodontists.

## 2. Materials and Methods

This study has been conducted in accordance with the STROBE guidelines for the presentation of cross-sectional studies [15]. Ethical approval for the study was obtained from James Cook University’s human research ethics committee (HREC Approval #H7701, 24 April 2019). The study was conducted in Australia, with convenience sampling used to recruit Australian periodontists. Information about practices with periodontists was obtained from public domain websites and this information was further verified through telephone conversation. The surveys were created electronically using SurveyMonkey^TM^ (San Mateo, CA, USA) and administered through email links sent to the participating practices. Although all Australian periodontists were invited to participate in this study, the information page at the beginning of the survey explicitly stated that the participation was voluntary and completion of the survey implied consent. The survey could only be filled out once by each participating periodontist. All collected survey data was treated as confidential and anonymous. Data collection was completed between November 2019 and February 2020. Inclusion criteria included all periodontists currently registered with the Australian Health Practitioner Regulation Agency (AHPRA) and actively practicing as a periodontist in Australia. Exclusion criteria included those without registration or not actively practicing.

### 2.1. Questionnaire Design

A five-part questionnaire was developed based on previously validated and published surveys in Australia [2], Sweden [14] and the USA [1]. The five sections within the questionnaire included demographics; implant history; prevalence and aetiology of peri-implant diseases; treatment and management of peri-implant diseases; training and education surrounding peri-implant disease. This survey was validated in its online format by two prominent Australian periodontists associated with post-graduate dental schools in Australia (University of Queensland and University of Sydney). Most questions were close-ended multiple choice questions, with an option for open-ended responses where appropriate. Other response types included a sliding percentage scale (0–100) and a ranking matrix which required practitioners to grade options from most common to least. The survey consisted of 26 questions in total and took an average of seven minutes to complete.

### 2.2. Statistical Analyses

According to the Dental Board of Australia’s December 2019 registration data table, there were 237 registered periodontists in Australia. Based on this figure, a required sample size of 93 periodontists was calculated, accounting for a 95% confidence interval, estimated portion of 50% and an 8% margin of error. Descriptive statistics (counts and percentages) were primarily used for analysis. Pearson’s chi-squared test (χ^2^) was used to evaluate univariate associations between categorical outcomes and other variables. Statistical significance was set at *p*-values < 0.05, with a 95% confidence interval (CI). SPSS IBM Statistics version 25 for Windows package (Chicago IL, USA) was used to analyse the data.

## 3. Results

Of the 237 registered periodontists in Australia, 108 responded to the survey. Of these 108 responses, 99 practitioners completed the entire survey, resulting in a completion rate of 91.7% and a response rate of 41.8%. Data collected from 8 practitioners who failed to complete the survey were excluded from the analysis.

### 3.1. Demographics

Most respondents in this study identified as male (73.7%) and were aged 35 to 44 years (48.5%). Most practitioners completed their periodontal training from either the University of Melbourne (29.3%) or the University of Queensland (28.3%). A little more than a quarter of practitioners had been practicing periodontists for 6–10 years (27.3%), while a little less than a quarter had done it for 11–15 years (24.2%). (Table 1)

### 3.2. Implant History

All but 2 practitioners reported that they placed dental implants (98%), with more than a quarter doing so for 11–15 years (28.9%). Almost three-quarters of practitioners reported that they placed either 1–10 implants (38.5%) or 11–20 implants (36.5%) on average per month. A significant association was found between increased years placing implants to implants placed/month (χ^2^ = 37.894, df = 16, *p* = 0.002) (Table 1).

### 3.3. Prevalence and Aetiology of Peri-Implant Diseases

**Figure 1:**
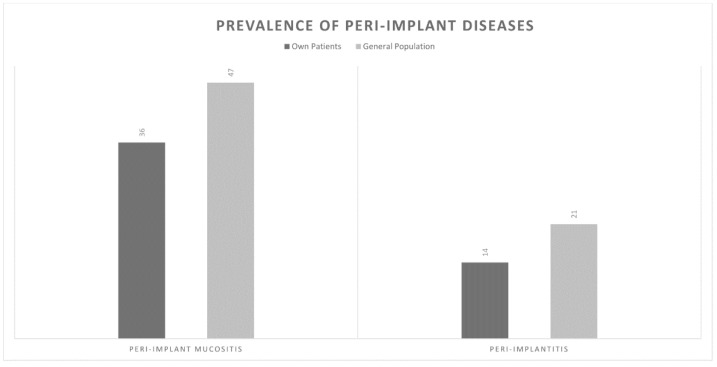
Estimated Prevalence of Peri-implant Diseases in the Australian population.

Each periodontist was asked to estimate the percentage of peri-implant mucositis and peri-implantitis amongst their patients and the general Australian population on a percentage scale of 0–100. Periodontists predicted that approximately 36% and 14% of their own patients suffer from peri- implant mucositis and peri-implantitis, respectively. They also predicted that the corresponding figures in the general Australian population were 47% and 21%, respectively. Furthermore, practitioners estimated that 12% of implants eventually need to be removed due to peri-implantitis (Figure 1).

**Figure 2:**
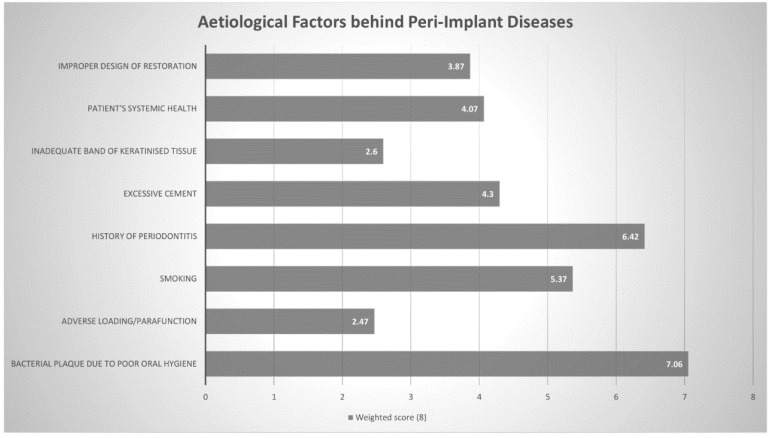
Aetiological Factors behind Peri-implant Diseases Weighted Score out of 8 (Higher = more commonly chosen).

Subsequently, practitioners were then asked to rank eight aetiological factors from most likely to contribute towards peri-implant pathology to least likely. All factors were then given a weighted average score out of 8 (higher meaning more commonly chosen). The three most commonly chosen factors were bacterial plaque accumulation (7.06), history of periodontitis (6.42) and smoking (5.37) (Figure 2 and Table 2).

### 3.4. Management of Peri-Implant Diseases

Concerning post-operative systemic antibiotics, practitioners stated that they would use them for 7% and 72% of peri-implant mucositis and peri-implantitis cases, respectively. Amoxicillin and Metronidazole combination was the most commonly selected antibiotic(s) that was utilised as first-line management for peri-implant diseases (41.8%), followed by azithromycin (26.5%). (Table 3) A significant association was found between practitioners who chose the combination amoxicillin and metronidazole and those who had been placing implants for over 20 years (χ^2^ = 56.842, df = 20, *p* < 0.001), as well as those who placed more than 50 implants/month (χ^2^ = 35.900, df = 20, *p* = 0.016).

**Figure 3:**
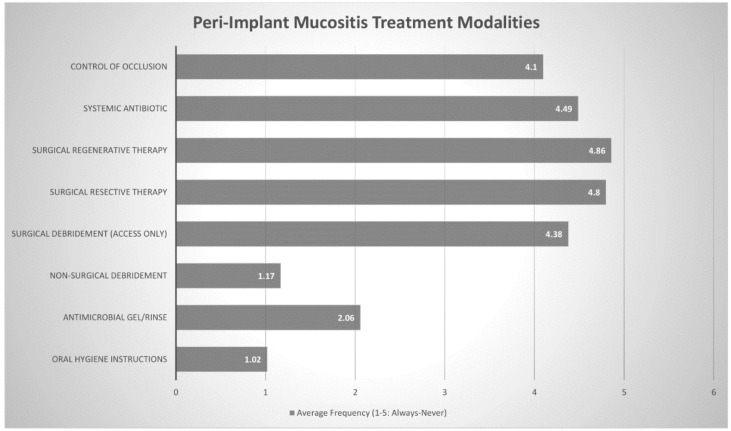
Peri-implant Mucositis Treatment Modalities Frequency (Always (1) through to Never (5)).

**Figure 4:**
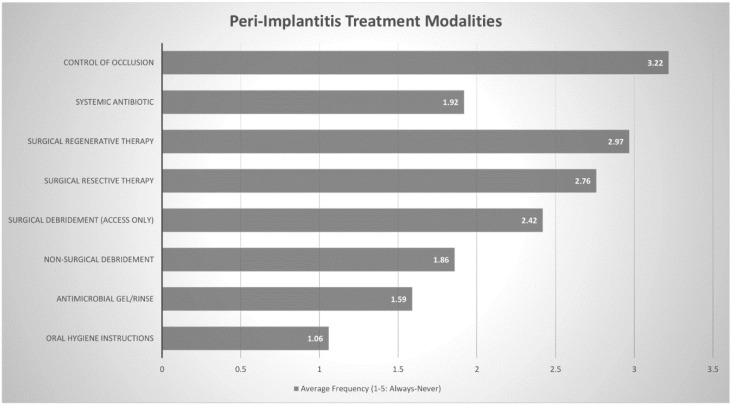
Peri-implantitis Treatment Modalities Frequency (Always (1) through to Never (5)).

Practitioners were also asked to rank the frequency of (always, often, sometimes, rarely or never) various proposed treatment modalities for peri-implant pathology. These modalities were then assigned a numerical value (ranging from 1 through to 5) to determine the most commonly chosen treatment protocols. The three most commonly chosen treatment modalities for peri-implant mucositis were oral hygiene instructions (1.02), nonsurgical debridement (1.17) and antimicrobial gel/rinse (2.06). All other modalities received scores between 4 and 5, suggesting that they were rarely-to-never used to treat peri-implant mucositis. Regarding treatment of peri-implantitis, oral hygiene instructions (1.06), antimicrobial gel/rinse (1.59) and nonsurgical debridement (1.86) were still the three most commonly chosen responses, although the other options were not so uncommon. Systemic antibiotics (1.92), surgical debridement (2.42), surgical resective therapy (2.76) and surgical regenerative therapy (2.97) all received scores around 2–3, suggesting that they were used often or sometimes. (Figure 3 and Figure 4 + Table 4 and Table 5)

**Figure 5:**
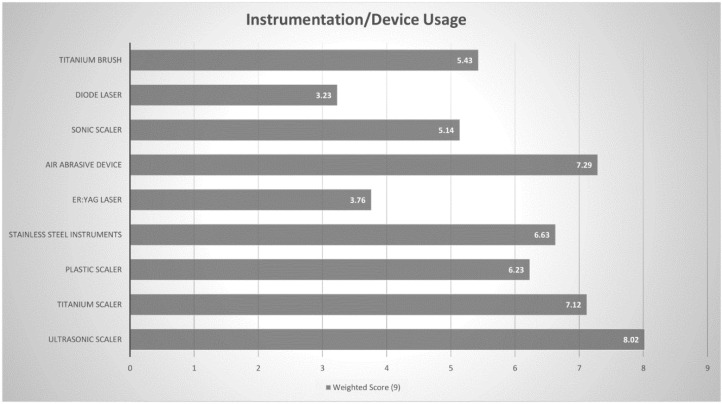
Instrumentation/Device Weighted Score (Out of 9).

**Figure 6:**
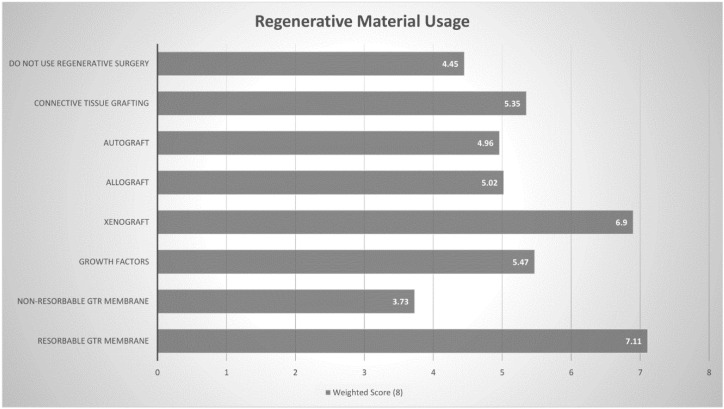
Regenerative Material Weighted Score (Out of 8).

Details on the instrumentation used for the debridement of implant surfaces in peri-implant diseases were sought through a rank question, in which a weighted score was determined out of 9 (Higher meaning more commonly used). The three most commonly chosen instruments/devices were ultrasonic scaler (8.02), air abrasive device (7.29) and a titanium scaler (7.12). (Figure 5 and Table 6). Similarly, practitioners were requested to rank the regenerative materials they used to treat peri- implant diseases and a weighted score out of 8 was calculated. The three most commonly chosen regenerative materials were resorbable GTR (guided tissue regeneration) membrane (7.11), xenograft (6.90) and growth factors (5.47) (Figure 6 and Table 7).

Regarding follow-up visits, most practitioners stated that a patient who has been successfully treated for peri-implant disease should be seen every 3 months (56.1%) or 6 months (37.8%). There was no significant association found between choice of follow-up visits and either years placing implants (χ^2^ = 9.907, df = 12, *p* = 0.607) or number of implants placed per month (χ^2^ = 16.884, df = 12, *p* = 0.155). Most practitioners would wait 6 months after successful treatment before conducting a radiographic evaluation of the treated site (56.6%). Practitioners who had greater experience with implants, through both years placing them (χ^2^ = 46.73, df = 20, *p* = 0.001) and implants placed/month (χ^2^ = 63.587, df = 20, *p* < 0.001) were significantly more likely to report a longer radiographic evaluation period of 12 months. (Table 3)

### 3.5. Training and Education Surrounding Peri-Implant Disease

More than three-quarters of practitioners opined that their current management of peri-implant disease was moderately effective (76.8%) and almost all practitioners (96.0%) had updated their training in this field within the last 2 years. Although there was no significant association between years placing implants (χ^2^ = 12.145, df = 8, *p* = 0.162) and self-assessment of effectiveness, those who placed more implants/month (χ^2^ = 26.326, df = 8, *p* = 0.030) were significantly more likely to report that their management was only mildly effective as opposed to moderately effective. Practitioners who had been placing implants for 11–15 years (χ^2^ = 18.311, df = 4, *p* = 0.008) and those placing 21–30 implants/month (χ^2^ = 19.396, df = 4, *p* = 0.004) were significantly more likely to report that they hadn’t updated their training in this area in the last two years. Despite this, more than nine-tenths of practitioners (90.9%) expressed that there should be increased training and awareness surrounding management of peri-implant disease. (Table 8) While implants placed/month (χ^2^ = 8.407, df = 4, *p* = 0.073) did not have a significant impact on increasing awareness, those who had been placing implants for 11–15 years (χ^2^ = 24.447, df = 4, *p* = 0.001) were more likely to report that increased awareness in this field was not required.

## 4. Discussion

Inconsistent definitions, lack of clear clinical parameters and various reporting methods have led to variable reports on the prevalence of peri-implant diseases [16,17]. The definitions set out by the WWP in 2017 clarified clinical cut-off points for peri-implant pathologies for both everyday clinical practice and epidemiological studies [3,6,7,18]. Applying these parameters to the best of their ability, Cosgarea et al. reported the prevalence of peri-implant mucositis to be 46.8% and peri-implantitis to be 18.5 [19] and 19.83% [18] for peri-implantitis [20]. In this study, Australian periodontists reported a disease prevalence of 47% and 21% for peri-implant mucositis and peri-implantitis, respectively. Although these figures closely match those reported previously, [20] practitioners believed that the disease prevalence in their own patients was significantly lower, at 36% and 14% respectively. Papathanasiou et al. also found that American periodontists felt that the general population had a higher disease prevalence than their own patients. They attributed this finding to possible psychologic factors and response bias [1]. This discrepancy may also be due to perceived differences in definition of peri-implant disease between epidemiological studies and practitioners in their clinical settings.

Dental Implant therapy is associated with a high success rate and this has been reported by several authors, [1,2,3,4,5] including Adler et al. who reported a cumulative implant survival rate of 82.6% over a 9–15-year follow-up period [4]. Patients in the study conducted by Adler et al. underwent dental therapy in Stockholm, Sweden (1999–2005) [4]. Participants in this study estimated that approximately 12% of implants needed to be removed due to peri-implantitis in the Australian population. While biological complications are most prevalent, there are other complications which can further affect the implant survival rate. Accounting for other complications, Australian periodontists seem to be experiencing similar cumulative implant survival rates as that reported by Adler et al. [4].

In the 2017 WWP, Heitz-Mayfield et al. [6] and Schwarz et al. [7] elaborated on potential risk factors/indicators with substantial evidence for peri-implant diseases. These included poor plaque control, history of periodontitis, smoking and diabetes. In the current study, Australian periodontists identified bacterial plaque, history of periodontitis and smoking as the three most significant aetiological/predisposing factors behind peri-implant pathology. Several cross-sectional studies have demonstrated that biofilm accumulation is associated with the development of peri-implant mucositis lesions [21,22,23]. Roos-Jansaker et al. studied 218 patients with 999 implants in function over a period of 9- to-14 years, reporting a significant association between plaque scores and peri-implant mucositis [22]. The progression from peri-implant mucositis to peri-implantitis has been explored in numerous studies [24,25]. Costa et al. conducted a retrospective study of 80 patients initially suffering from peri- implant mucositis. Over a five-year period, they found that the incidence of peri-implantitis was lower (18%) in subjects enrolled in a regular maintenance program than those without (43%) [25]. Roccuzzo et al., [26] Monje et al. [27] and Kassebaum et al. [28] reported that patients who complied to a regular maintenance therapy following implant therapy were significantly less likely to be diagnosed with peri- implantitis, over a period of 10 years, 2 years and 3.8 years, respectively.

Periodontitis is one of the most prevalent diseases in the world, [28] and it has been found to be associated with peri-implantitis. Karoussis et al. [29] and Roccuzzo et al. [26,30] conducted 10-year longitudinal studies, finding that peri-implantitis incidence in individuals with a history of periodontitis was significantly higher. Roccuzzo et al. also reported that treatment of peri-implantitis was more time-consuming in such patients [26,30].

It has been well established that smoking is strongly associated with periodontitis, attachment loss and tooth loss [7]. It is thought that this effect translates to peri-implant tissues as well. Karoussis et al. found that 18% of all implants in smokers developed peri-implantitis, compared to 6% in nonsmokers, over a period of ten years [29]. However, several cross-sectional studies have also reported that smokers are not at higher risk for developing peri-implantitis [31,32,33]. These could potentially be due to confounding factors, but nonetheless, Schwarz et al. concluded that there is currently no conclusive evidence that smoking constitutes a risk factor for peri-implantitis [7].

In 2011, Mombelli et al. conducted a review in which they concluded that the combination of metronidazole and amoxicillin has the potential to overcome a wide range of pathogens often associated with peri-implant disease [34]. Amoxicillin, [35] metronidazole [36] and azithromycin [37] have all proven to be useful adjuncts in the treatment of peri-implant disease [12]. Despite their short-term efficacy, there does not appear to be strong evidence that these effects are sustained in the longer term, [38] and antimicrobial resistance has also been detected in peri-implant biofilms [39]. The combination of amoxicillin and metronidazole was the most commonly chosen first-choice antibiotic regimen amongst Australian periodontists in this study, particularly amongst those who had been placing implants for more than 20 years or 50+ implants/year.

Practitioners in this study found that oral hygiene instructions, nonsurgical debridement and antimicrobial gel/rinse were effective enough treatment modalities to counter peri-implant mucositis. This notion was supported by Derks et al. who concluded that nonsurgical approaches with thorough mechanical debridement appear to be effective approach for peri-implant mucositis, but outcomes are more guarded in the management of peri-implantitis [16]. Interestingly, Australian periodontists in our study, were increasingly likely to use surgical approaches and systemic antibiotics to treat peri- implantitis lesions. This may be due to the fact that while nonsurgical treatment is still often performed as part of initial therapy, it has been reported to only be moderately effective in the management of peri-implantitis [40,41]. Surgical techniques offer more predictable outcomes, [42] through improved access and the possibility for resective and regenerative procedures, [43] with several studies reporting positive outcomes [44,45]. Australian periodontists report using surgical techniques often—sometimes to manage peri-implantitis lesions.

Several instruments have been used to debride affected implant surfaces in both surgical and nonsurgical approaches [46]. Ultrasonic devices and titanium curettes [11] have been favoured among UK [2] and American periodontists, [1] in line with Australian periodontists. Air-abrasive devices are also popular amongst Australian periodontists, although there is little evidence supporting their enhanced cleaning efficacy in the long term, as compared to other instruments [47,48]. Surgical regenerative therapy was the least likely treatment protocol used by periodontists treating peri-implant lesions and is rarely utilised by Australian periodontists. Amongst those that use surgical approaches for management of peri-implantitis lesions, the most common regenerative procedure employed was GTR membrane alone, despite some studies demonstrating minimal benefit of barrier membranes when compared to bone-grafting alone [49,50].

Supportive maintenance therapy with regular reviews is essential for all patients treated for peri- implant disease [13] with most Australian periodontists considering a 3-month follow-up as most preferred. A majority of practitioners conducted a radiographic evaluation of the treated site 6- months after treatment. Practitioners with greater experience through years placing implants and implants placed/month were more likely to conduct a radiographic evaluation at 12 months, suggesting that 6 months may be too early to expect radiographic changes in the treated site. Papathanasiou et al. reported similar findings in America, where the majority of practitioners also reported 3-month follow ups and a 6-month radiograph, although they found that less experienced practitioners were more likely to conduct a radiographic evaluation sooner than 6 months [1].

Although more than three-quarters of practitioners in this study rated their management of peri- implant diseases as moderately effective, it was found that those placing more implants/month were significantly more likely to report their management as mildly effective. The increase in implants placed likely leads to an increase in the number of failures seen, particularly peri-implant diseases, and conditions of varying severity may lead these clinicians to be more cautious in their self- assessment. All but four practitioners reporting that they had updated their training around peri- implant pathology in the last two years and upwards of 90% of practitioners suggested that greater awareness surrounding the management of peri-implant disease is warranted. These figures reflect the continuing uncertainty in this field, with a gold standard management protocol yet to be established, [10,11,12,13] with one participant aptly stating that “unfortunately, most of the research just tells us what doesn’t work.” While this study has shed light on an increasingly important matter, it does have its limitations, particularly due to its response rate. Although it has a higher response rate than similar studies in the USA [1] and the UK, [2] it still falls short of 50% of all eligible periodontists in Australia. While this remains a significant barrier, this study has utilised other means to improve the quality of data, including the use of an electronic format. The electronic format has enabled the collection of accurate and versatile data, with precise prevalence percentages along with a ranking system for aetiology, treatment and instrumentation reported as weighted scores. This electronic format coupled with clear definitions and clinical parameters in accordance with the 2017 WWP have enabled the creation of reliable baseline data for future research in this field.

## 5. Conclusions

This study demonstrates that Australian periodontists acknowledge the increasing prevalence of peri-implant diseases and frequently deal with them in their routine practice. Although most periodontists agreed that the most common aetiological factor behind peri-implant diseases is bacterial accumulation, there was significant heterogeneity regarding treatment protocols. Due to the lack of a standard therapeutic protocol, there appears to be significant empirical use of therapeutic modalities. Despite this, most periodontists believe that their management is moderately effective and continue to update their training regularly. These results allow general dental practitioners to consider various approaches employed by periodontists in management of peri-implant diseases and scaffold their treatment approaches. Future studies elaborating on successful treatment protocols are needed as they become widely accepted and adopted by periodontists. 

## Figures and Tables

**Table 1:** Practitioners’ Demographics and Implant History.

	Frequency (n)	Percentage (%)
**Gender**		
Male	73	73.74
Female	26	26.26
**Age**		
26–34 years old	1	1.01
35–44 years old	48	48.48
45–54 years old	18	18.18
55–64 years old	27	27.27
Over 65 years	5	5.05
**Post-Graduate Education**		
Griffith University	6	6.06
University of Adelaide	12	12.12
University of Melbourne	29	29.29
University of Queensland	28	28.28
University of Sydney	11	11.11
University of Western Australia	9	9.09
Outside Australia	4	4.04
**Periodontist Experience**		
2–5 years	8	8.08
6–10 years	27	27.27
11–15 years	24	24.24
16–20 years	15	15.15
21–25 years	6	6.06
26–30 years	12	12.12
31–35 years	3	3.03
36–40 years	4	4.04
**Implant Placement**		
Yes	97	97.98
No	2	2.02
**Years Placing Implants**		
1–5 years	4	4.12
6–10 years	23	23.71
11–15 years	28	28.87
15–20 years	18	18.56
More than 20 years	24	24.74
**Implants Placed/Month**		
1–10	37	38.54
11–20	35	36.46
21–30	17	17.71
31–40	0	0
41–50	2	2.08
50+	5	5.21

**Table 2:** Aetiological Factor Ranking: weighted score out of 8 (higher = more commonly chosen).

Aetiological Factor/Rank	1	2	3	4	5	6	7	8	N/A	Weighted Score (8)
**Bacterial plaque due to poor oral hygiene**	56.12%	18.37%	15.31%	3.06%	4.08%	1.02%	0%	2.04%	0%	7.06
**Adverse loading/Parafunction**	2.04%	3.06%	0%	6.12%	10.20%	18.37%	12.24%	40.82%	7.14%	2.47
**Smoking**	7.14%	21.43%	27.55%	11.22%	14.29%	14.29%	4.08%	0%	0%	5.37
**History of Periodontitis**	29.59%	28.57%	16.33%	15.31%	4.08%	4.08%	0%	2.04%	0%	6.42
**Excessive Cement**	3.03%	13.13%	8.08%	15.15%	28.28%	16.16%	12.12%	4.04%	0%	4.30
**Inadequate band of keratinised tissue**	2.04%	0%	0%	11.22%	12.24%	12.24%	36.73%	23.47%	2.04%	2.60
**Patient’s systemic health**	0%	5.05%	20.20%	20.20%	13.13%	18.13%	19.19%	4.04%	0%	4.07
**Improper design of restoration**	0%	10.10%	12.12%	18.18%	14.14%	14.14%	16.16%	13.13%	2.02%	3.87

**Table 3:** Practitioners’ Clinical Management of Peri-Implant Diseases.

	Frequency (n)	Percentage (%)
**Antibiotic Choice**		
Amoxicillin	21	21.43
Amoxicillin + Clavulanic Acid	3	3.06
Amoxicillin + Metronidazole	41	41.84
Azithromycin	26	26.53
Metronidazole	4	4.08
**Clinical Recall Frequency**		
Every 3 months	55	56.12
Every 6 months	37	37.76
Every 12 months	1	1.02
**Radiographic Evaluation**		
Immediately	5	5.05
3 months	10	10.10
6 months	56	56.57
9 months	2	2.02
12 months	22	22.22

**Table 4:** Peri-implant Mucositis Treatment Modalities: Average Frequency (Always (1) through to Never (5)).

Frequency/Therapeutic Modality	Always (1)	Often (2)	Sometimes (3)	Rarely (4)	Never (5)	Average Frequency (1–5)
Oral Hygiene Instructions	97.98%	2.02%	0%	0%	0%	1.02
Antimicrobial gel/rinse	43.16%	23.16%	23.16%	5.26%	5.26%	2.06
Nonsurgical debridement	88.89%	8.08%	0%	3.03%	0%	1.17
Surgical debridement (access only)	0%	4.49%	12.36%	23.60%	59.55%	4.38
Surgical resective therapy	0%	0%	5.43%	8.70%	85.87%	4.80
Surgical regenerative therapy	1.11%	0%	0%	10.00%	88.89%	4.86
Systemic Antibiotic	0%	0%	14.13%	22.83%	63.04%	4.49
Control of Occlusion	0%	10.87%	22.83%	11.96%	54.35%	4.10

**Table 5:** Peri-implantitis Treatment Modalities: Average Frequency (Always (1) through to Never (5)).

Frequency/Therapeutic Modality	Always (1)	Often (2)	Sometimes (3)	Rarely (4)	Never (5)	Average Frequency (1–5)
Oral Hygiene Instructions	97.96%	0%	0%	2.04%	0%	1.06
Antimicrobial gel/rinse	60.00%	27.37%	6.32%	6.32%	0%	1.59
Nonsurgical debridement	58.33%	18.75%	2.08%	19.79%	1.04%	1.86
Surgical debridement (access only)	13.27%	51.02%	21.43%	9.18%	5.10%	2.42
Surgical resective therapy	4.21%	44.21%	27.37%	20%	4.21%	2.76
Surgical regenerative therapy	2.08%	39.58%	27.08%	21.88%	9.38%	2.97
Systemic Antibiotic	43.88%	30.61%	19.39%	2.04%	4.08%	1.92
Control of Occlusion	12.77%	15.96%	20.21%	38.30%	12.77%	3.22

**Table 6:** Debridement instrumentation/devices ranking: weighted score out of 9 (higher = more commonly chosen).

Instrumentation/Rank	1	2	3	4	5	6	7	8	9	N/A	Weighted Score (9)
Ultrasonic Scaler	40.40%	33.33%	6.06%	11.11%	1.01%	0%	0%	0%	1.01%	7.07%	8.02
Titanium Scaler	19.39%	15.31%	10.20%	10.20%	8.16%	5.10%	1.02%	0%	0%	30.61%	7.12
Plastic Scaler	3.03%	4.04%	16.16%	16.16%	14.14%	3.03%	0%	0%	0%	43.43%	6.23
Stainless Steel Instruments	3.03%	19.19%	23.23%	17.17%	17.17%	0%	1.01%	0%	0%	19.19%	6.63
Er:YAG Laser	0%	2.06%	1.03%	0%	4.12%	6.19%	5.15%	5.15%	2.06%	74.23%	3.76
Air Abrasive Device	32.32%	9.09%	12.12%	8.08%	5.05%	4.04%	4.04%	1.01%	0%	24.24%	7.29
Sonic Scaler	0%	7.14%	5.10%	3.06%	2.04%	9.18%	10.20%	0%	0%	63.27%	5.14
Diode Laser	0%	0%	5.10%	2.04%	1.02%	5.10%	2.04%	15.31%	5.10%	64.29%	3.23
Titanium Brush	0%	9.18%	14.29%	7.14%	7.14%	10.20%	4.08%	0%	5.10%	42.86%	5.43

**Table 7:** Regenerative materials ranking: weighted score out of 8 (higher = more commonly chosen).

Regenerative Material/Rank	1	2	3	4	5	6	7	8	N/A	Weighted Score (8)
Resorbable GTR Membrane	41.05%	29.47%	13.68%	0%	4.21%	0%	1.05%	0%	10.53%	7.11
Nonresorbable GTR Membrane	0%	1.10%	1.10%	10.99%	4.40%	6.59%	7.69%	1.10%	67.03%	3.73
Growth Factors	0%	12.90%	11.83%	11.83%	8.60%	3.23%	0%	0%	51.61%	5.47
Xenograft	45.26%	20.00%	9.47%	0%	0%	3.16%	0%	5.26%	16.84%	6.90
Allograft	3.19%	10.64%	10.64%	7.45%	3.19%	4.26%	9.57%	0%	51.06%	5.02
Autograft	2.20%	8.79%	12.09%	9.89%	12.09%	13.19%	0%	0%	41.76%	4.96
Connective Tissue Grafting	2.11%	11.58%	27.37%	31.58%	8.42%	2.11%	3.16%	1.05%	12.63%	5.35
Do Not Use Regenerative Surgery	6.45%	0%	0%	5.38%	3.23%	0%	5.38%	3.23%	76.34%	4.45

**Table 8:** Practitioners’ Attitude Towards Training.

**Efficacy of Current Management**		
Ineffective	0	0
Mildly effective	21	21.21
Moderately effective	76	76.77
Very effective	2	2.02
**Updated Training in Last 2 Years**		
Yes	95	95.96
No	4	4.04
**Increased Awareness and Training Warranted**		
Yes	90	90.91
No	9	9.09

## Abbreviations and Acronyms

WWP	World Workshop on Periodontology
USA	United States of America
UK	United Kingdom
GTR	Guided Tissue Regeneration
